# A Multi-Enzymatic Cascade Reaction for the Stereoselective Production of γ-Oxyfunctionalyzed Amino Acids

**DOI:** 10.3389/fmicb.2016.00425

**Published:** 2016-04-07

**Authors:** Junichi Enoki, Jaqueline Meisborn, Ann-Christin Müller, Robert Kourist

**Affiliations:** Faculty of Biology and Biotechnology, Junior Research Group for Microbial Biotechnology, Ruhr-University BochumBochum, Germany

**Keywords:** enzyme reaction, isoleucine dioxygenase, dynamic kinetic resolution, multi-enzyme cascade reaction, amino acids, asymmetric oxidation

## Abstract

A stereoselective three-enzyme cascade for synthesis of diasteromerically pure γ-oxyfunctionalized α-amino acids was developed. By coupling a dynamic kinetic resolution (DKR) using an *N*-acylamino acid racemase (NAAAR) and an L-selective aminoacylase from *Geobacillus thermoglucosidasius* with a stereoselective isoleucine dioxygenase from *Bacillus thuringiensis*, diastereomerically pure oxidized amino acids were produced from racemic *N*-acetylamino acids. The three enzymes differed in their optimal temperature and pH-spectra. Their different metal cofactor dependencies led to inhibitory effects. Under optimized conditions, racemic *N*-acetylmethionine was quantitatively converted into L-methionine-(*S*)-sulfoxide with 97% yield and 95% *de*. The combination of these three different biocatalysts allowed the direct synthesis of diastereopure oxyfunctionalized amino acids from inexpensive racemic starting material.

## Introduction

Cascade and one-pot reactions represent an exciting development in *White Biotechnology* (Ricca et al., 2011). The concept of performing multi-step syntheses in one-pot, despite not being very new, has received increased attention in the past years. From an environmental point of view, cascades represent a very promising approach, mainly due to the avoidance of intermediate extraction and purification steps, resulting in a significant reduction of both waste and production costs on industrial scale. There are, however, some technological and scientific challenges to be overcome *en route* to industrial scale implementation of cascades. One of the most common challenges for the practicability of a cascade reaction is the combination of biocatalysts from different sources, which often have different optimal reaction conditions and show undesired side reactions.

Hydroxy amino acids represent an important class of natural products and bioactive ingredients. In the last years, several amino acid hydroxylases have been isolated and characterized. (2*S*,3*R*,4*S*)-4-hydroxyisoleucine (4-HIL), originally isolated from fenugreek seeds, exhibits an antidiabetic and anti-obesity activity that makes it an attractive target for the production of functional foods (Fowden et al., 1973; Smirnov et al., 2012). L-*Threo*-3-hydroxyaspartic acid (L-THA) has broad clinical and material utility as an antimicrobial agent against various microorganisms (Ishiyama et al., 1975), as an inhibitor of glutamate transporters (Kidd and Isaac, 2000) and as a functional moiety of polymethacrylamide polymers (Sanda et al., 1999). Viomycin, a tuberactinomycin family of non-ribosomal peptide antibiotics, contains (2*S*,3*S*)-hydroxyarginine which is produced from L-arginine by an amino acid dioxygenase VioC (Helmetag et al., 2009). The enzymatic hydroxylation of proline (Katsumata and Yokoi, 1994) and isoleucine (Kodera et al., 2013) has found industrial application. Moreover, these chiral oxyfunctionalized amino acids are useful as building blocks for drugs (Hibi et al., 2011).

**Scheme 1:**
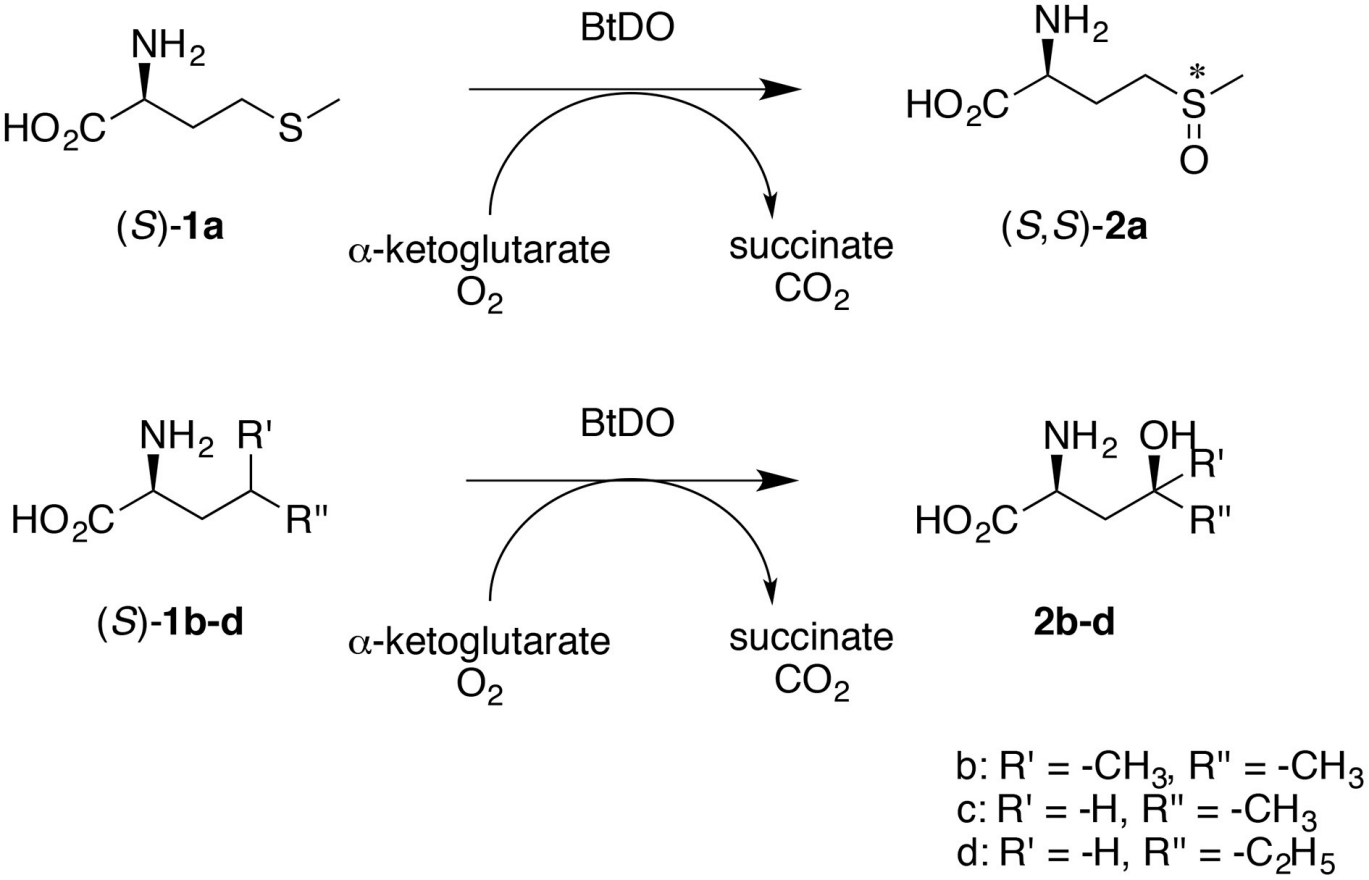
**BtDO catalyzes the stereoselective γ-specific oxidation of aliphatic L-amino acids such as L-methionine (above) and several branched-chain amino acids (below)**.

Natural amino acid hydroxylation is carried out by Fe(II)/α-ketoglutarate dependent enzymes. One of the most promising is the L-isoleucine dioxygenase from *Bacillus thuringiensis* (BtDO), which catalyzes the enantioselective hydroxylation of several hydrophobic amino acids with specificity for the γ-position (Kodera et al., 2009; Hibi et al., 2011; Ogawa et al., 2011; Smirnov et al., 2013). Interestingly, BtDO also catalyzes the highly enantioselective sulfoxidation of L-methionine (Scheme 1; Hibi et al., 2013b). Recently, a δ-specific leucine dioxygenase from *Nostoc punctiforme* was reported (Hibi et al., 2013a). A general feature of these Fe(II)/α-ketoglutarate dependent amino acid hydroxylases is their strict specificity for the L-enantiomer. While several proteinogenic L-amino acids are readily available from fermentation, many non-proteinogenic amino acids (as well as the proteinogenic amino acid L-methionine) are considerably cheaper in racemic form. Therefore, for the hydroxylation of non-canonical amino acids, application of amino acid dioxygenases requires either the use of costly optically pure starting material or requires to resolve the racemate. The latter limits the yield to 50% and requires the separation of the hydroxylated enantiomer from the remaining D-amino acid.

**Scheme 2:**
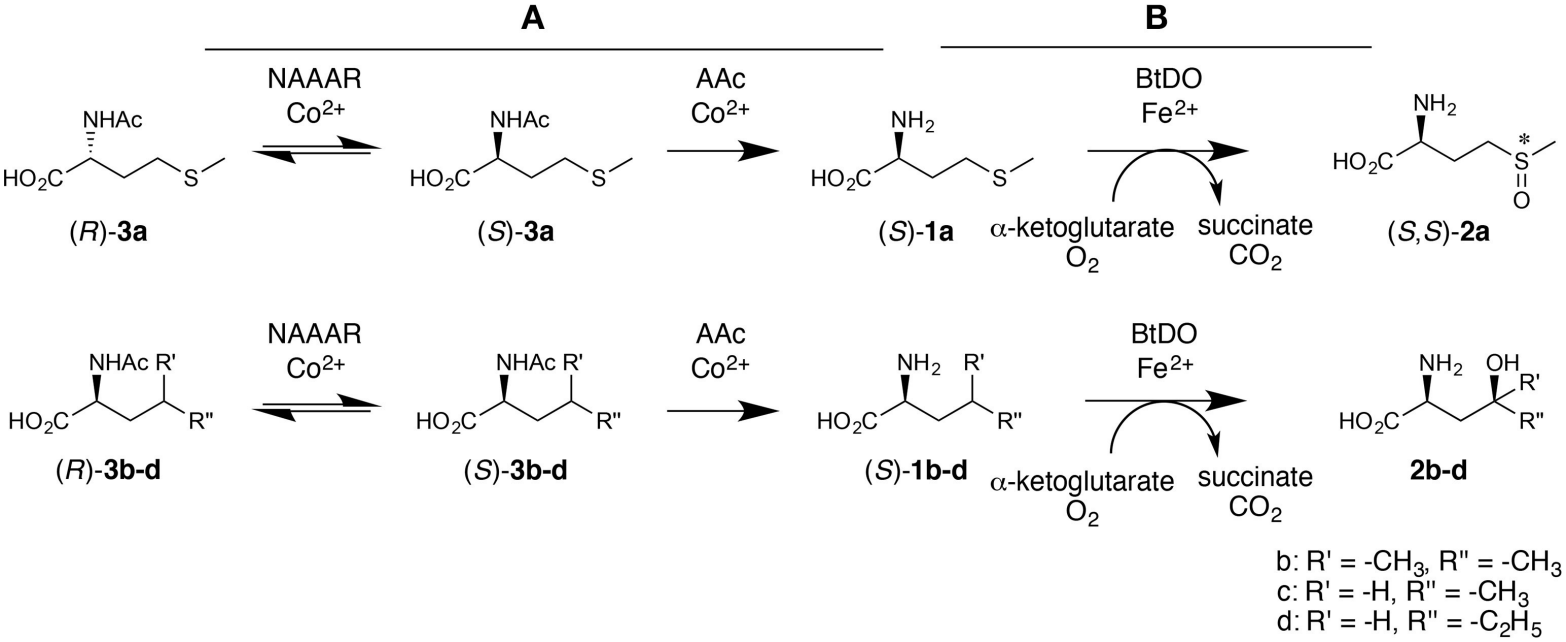
**Multi-enzyme cascade reaction combining racemization and enantioselective hydrolysis of ***N***-acylamino acids with enantioselective oxidation**. Dynamic kinetic resolution step by AAc and NAAAR **(A)**, and L-amino acid selective oxyfunctionalization by BtDO **(B)**. The approach requires mutual compatibility of the three biocatalysts regarding temperature, pH and, most importantly, the metal cofactors.

In the enantioselective conversion of racemates, dynamic kinetic resolution (DKR) has emerged as an efficient strategy to increase the yield to a 100% maximum production of chiral compounds from racemic starting materials and facilitate the down-stream processing (May et al., 2002). Since it has been recently shown that amino acid dioxygenases can be very efficiently applied in multi-enzyme cascade reactions (Hibi et al., 2015), we reasoned that a combination of amino acid dioxygenases with simultaneous racemization in a one-pot reaction might circumvent the drawback of kinetic resolutions. A successful DKR, however, requires a rigorous substrate selectivity: The substrate should not be racemized rapidly but the product must not be catalyzed. Racemization by *N*-acylamino acid racemases (NAAAR) is expected to avoid this problem as these enzymes do not convert free amino acids (Tokuyama, 2001). For instance, the combination of an NAAAR and an L-selective aminoacylase (AAc) with BtDO is expected to allow the one-pot synthesis of diastereomerically pure L-methionine-*S*-sulfoxide (*S*,*S*)-**2a** starting from *rac*-**3a** (Scheme 2). In this study, we established a novel multi-enzyme cascade approach for the production of chiral oxidized amino acids from racemic substrates. Application of BtDO yielded diastereomerically pure L-methionine-(*S*)-sulfoxide and γ-hydroxy amino acids.

## Materials and methods

### General

All chemicals were purchased from Sigma-Aldrich, TCI Organics and ALFA AESAR. ^1^H-NMR was measured using Bruker (Rheinstetten, Germany) DPX-400 NMR.

#### Cloning *btdo, aac*, and *naaar*

The gene of isoleucine dioxygenase (*btdo*) and the L-selective amino acylase (*aac*) were obtained from a genomic DNA of *B. thuringiensis* ATCC10792 (Hibi et al., 2011), *Geobacillus thermoglucosidasius* DSM2542 (Cho et al., 1987), respectively. A codon-optimized gene (compare Data Sheet 1) of NAAAR mutant (G291D/F323Y) from *Amycolatopsis* sp. Ts1-60 (*naaar*) (Baxter et al., 2012) was ordered as synthetic gene from Life Technologies (Darmstadt, Germany). Using these genomic DNA or synthetic gene as templates, gene amplification by PCR was carried out with Phusion® High-Fidelity DNA Polymerase (FINNZYMES OY, Espoo, Finland) under the following conditions: 30 s at 98°C; 35 cycles for 10 s at 98°C, 30 s at corresponding T_m_ (Table 1), and 45 s at 72°C; 10 min at 72°C; kept at 4°C. The PCR products were digested with corresponding endonucleases shown in Table 1 and cloned into an expression vector pET22b (*btdo* and *naaar*) or pET28b (*aac*) (Novagen, CA, USA), which has been digested with the same endonucleases. The constructed plasmid DNAs were introduced into *E. coli* BL21(DE3).

**Table 1:** **Primer sequences**.

**Gene**	**Direction (rest. site)**	**T_*m*_ (°C)**	**Primer sequence (5′ to −3′)^a^**
*btdo*	Fw (NdeI)	63	GACATATGAAAATGAGTGGCTTTAGCATAGAA
	Rv (XhoI)	65	GACTCGAGTTTTGTCTCCTTATAAGAAAATGTTAC
*aac*	Fw (NheI)	65	GCGCTAGCATGACCAATGAAGAGATCAAACGGC
	Rv (HindIII)	64	GCAAGCTTTTATGACGCTTCCGCCAATAATTTAAAC
*naaar* (G291D/F323Y)	Fw (NdeI)	63	ATCATATGAAACTGAGCGGTGTTGAAC
	Rv (XhoI)	63	ATCTCGAGTCCGCTACCAATCCAAACTTTTGC

a *The restriction sites are shown with under bar*.

#### Expression and purification of recombinant proteins

For expression, *E. coli* BL21(DE3) cells with a plasmid DNA containing the genes of recombinant protein were grown in 200 mL LB medium supplemented with the corresponding antibiotics (ampicillin 100 μg mL^−1^ for pET22 or kanamycin 30 μg mL^−1^ for pET28) at 37°C. After the OD_600_ reached 0.5, overexpression was induced by addition of IPTG (1 mM) and cultivated for overnight at 30°C. The cells were harvested by centrifugation (5000 x*g*, 20 min, 4°C) and washed with Tris-HCl buffer (20 mM, pH 7.4) containing NaCl (300 mM). After sonication on ice, the cell debris was removed by centrifugation (10,000 x*g*, 20 min, 4°C). The supernatant was applied to a Ni-affinity column and purified following the manufacturer's instructions. The buffer of the elution fraction was replaced with HEPES buffer (10 mM, pH 7.0) or potassium phosphate buffer (10 mM, pH 7.0) using 10 kDa Amicon Ultra Centrifugal Filter Units or by dialysis. The protein concentration was determined by the Bradford assay using BSA as standard.

#### Enzyme assays

Purified BtDO was used for sulfoxidation of (*S*)-**1a** or hydroxylation of (*S*)-**1b-d**. The reaction components were shown below: HEPES buffer (pH 7.0, 100 mM), (*S*)-**1a** (5 mM), α-ketoglutarate (10 mM), ascorbic acid (10 mM), FeSO_4_ (0.5 mM) with different concentrations of CoCl_2_ (0.1–1.6 mM). After 5 min incubation at 25°C, the reaction was initiated by adding 1.0 mg preincubated BtDO to a total volume of 1 mL. After 30 min reaction at 25°C, the enzyme was quenched by 2 M HCl. The product was quantified by high-performance liquid chromatography (HPLC) with *o*-phtalaldehyde (OPA) derivatization as detailed elsewhere (Cohen and Michaud, 1993).

DKR reactions with NAAAR and AAc were performed with HEPES (100 mM, pH 7.0), *rac*-**3a**-**d** (5 mM), AAc (80 μg mL^−1^) and of NAAAR (300 μg mL^−1^) with different combination of metal additive (0.2 mM CoCl_2_, 0.2 mM MnCl_2_, and 0.5 mM FeSO_4_) at 25°C or 40°C. Preincubation was performed at corresponding temperature for 5 min. The enzymatic reaction was quenched by addition of 2 M HCl. The product was detected by HPLC with OPA derivatization.

One-pot reactions with NAAAR, AAc and BtDO were performed with HEPES (100 mM, pH 7.0), *rac*-**3a** (5 mM), α-ketoglutarate (10 mM), L-ascorbate (10 mM), CoCl_2_ (0.2 mM), FeSO_4_ (0.5 mM), BtDO (1.0 mg mL^−1^), Ac (80 μg mL^−1^), and NAAAR (300 μg mL^−1^) at 25°C. Preincubation was performed at 25°C for 5 min. The enzymatic reaction was quenched by addition of 2 M HCl. The product was detected by HPLC with OPA derivatization.

#### Measurement of racemase activity by *in situ* ^1^H-NMR

The racemization activity NAAAR was analyzed by detecting the deuterium replacement of the α-proton of the substrate via ^1^H-NMR (Kourist et al., 2011). The reaction mixture was prepared in deuterium oxide as solvent with potassium phosphate buffer (100 mM, pH 7.0), *rac*-**3a**-**d** (5 mM) and NAAAR (300 μg mL^−1^) with different metal cofactors (0.2 mM CoCl_2_, 0.2 mM MnCl_2_, and 0.5 mM FeSO_4_. The chemical shift of the α-proton of **3a** was δ = 4.20–4.26.

#### HPLC analysis

Amino acids were determined by an AZURA high-performance liquid chromatography (HPLC) System (Knauer, Berlin, Germany) using the *o*-phtalaldehyde (OPA) derivatization method according to the instructions of the manufacturer (Cohen and Michaud, 1993). A NUCLEAODUR C18 Pyramid column (5 μm; 4.6 by 250 mm; Macherey-Nagel, Düren, Germany) was used for separation at 25°C. The mobile phase were acetonitrile (eluent A) and 10 mM sodium acetate buffer at pH 7.2 (eluent B), and the flow rate of the eluent was 0.8 mL min^−1^. The eluent gradients were 10% (vol/vol) A for 3 min, 10–40% A for 3–10 min, and 40% A for 10–18 min. The compounds were detected with a fluorescence detector at 355 nm and 450 nm for excitation and emission, respectively. The retention time of the analytes was as follows: **1a**, 13.1 min; **2a**, 11.2 min; **1b**, 13.7 min; **1c**, 13.1 min, **1d**, 13.9 min.

#### Chiral analysis

Enantiomeric excess (*ee*) or diastereomeric excess (*de*) of products was measured by an AZURA HPLC system (Knauer, Berlin, Germany) with chiral column CROWNPAK CR(+) (Daicel, Tokyo, Japan). The mobile phase was perchloric acid (16.3 g L^−1^, pH 1.0) and the flow rate of the eluent was 0.5 mL min^−1^ at 10°C for separation. The compounds were detected by 200 nm of UV absorption. The retention time of the compounds was determined to be: (*S*,*S*)-**2a**, 3.9 min; (*S*,*R*)-**2a**, 4.6 min; (*S*)-**1a**, 16.7 min; (*R*)-**1a**, 8.0 min.

## Results and discussion

### Combination of BtDo with direct racemization of the substrate by amino acid racemases

*B. thuringiensis* isoleucine dioxygenase BtDO is specific for the hydroxylation or sulfoxidation of aliphatic L-amino acids. BtDO does not convert the D-enantiomers (Hibi et al., 2011). Using racemic mixtures of amino acids as starting material, simultaneous racemization of amino acids coupled with the enantioselective oxyfunctionalization would be a direct approach as the dyamic kinetic resolution of amino acids. However, an important prerequisite for this DKR concept is that the racemising catalyst should not epimerize the diastereomerically pure products. We have cloned and expressed an amino acid racemase (AAR) from *Pseudomonas putida* (AAR) and an alanine racemase from *Geobacillus stearothermophilus* (AlaR). AAR has been reported to have a broad substrate spectrum (Ikeda et al., 2005), while AlaR is very specific for amino acids with a short hydrophobic side chain (Inagaki et al., 1986). Combination of these racemases with BtDO led to 100% conversion of racemic methionine into L-methionine-(*S*)-sulfoxide [(*S*,*S*)-**2a**]. To test whether the racemases also accept the reaction product of BtDO, we performed the enzymatic deuteration of the α-carbon atom of amino acids by *in situ* NMR experiments (Kourist et al., 2011). Both racemases catalyzed the α-epimerization of methionine sulfoxide (data not shown). Consequently, a direct DKR did not appear to be feasible since it would lead to a mixture of two diastereomers.

### Combination of BtDo with racemization and enantionselective acylation of the *N*-acetylamino acids

To avoid the epimerization, we then investigated the production of optically pure amino acids by the combination of an L-selective aminoacylase (AAc) with an NAAAR. NAAAR is specific for *N*-acylamino acids and does not accept free amino acids. AAc from *G. thermoglucosidasius* DSM2542 was previously purified from cell-free extracts of *G. thermoglucosidasius* and showed a high enantioselectivity toward several *N*-acylamino acids (Cho et al., 1987). Using the N-terminal amino acid sequence, we identified the open reading frame (Accession: CP012712 region: 384660.38544). The putative acylase (AAc) and mutant G291D/F323Y of the *N*-acyl amino acid racemase (NAAAR) were cloned and functionally expressed in *E. coli*. Cultivations in 200 mL scale yielded 8.2 mg of purified AAc and 5.3 mg of purified NAAAR. AAc hydrolyzed (*S*)-**3a** with a specific activity of 7.8 U mg^−1^ under the condition of 5 mM substrate concentration at 25°C with pH 7.0. Semi-quantitative activity tests of NAAAR toward of *N*-acylamino acids **3a** were performed by measuring the H-D exchange of the α-proton with deuterium oxide as a solvent.

### Mutual tolerance of the enzymes toward metal cofactors

**Figure 1:**
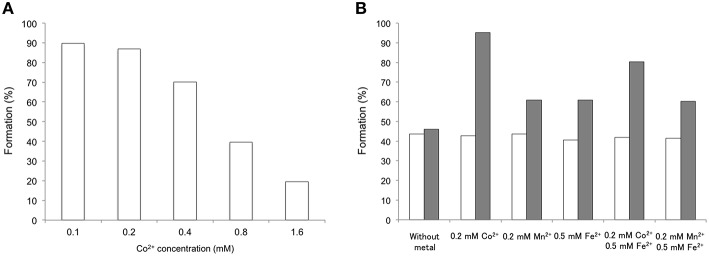
**Metal inhibition study on BtDO (A), AAc (B, white bars), and AAc/NAAAR (B, gray bars). (A)** HEPES buffer (pH 7.0, 100 mM), (*S*)-**1a** (5 mM), α-ketoglutarate (10 mM), ascorbic acid (10 mM), FeSO_4_ (0.5 mM), and purified BtDO (1.0 mg mL^−1^) with different concentrations of CoCl_2_ (0.1–1.6 mM). After 30 min. reaction at 25°C, the enzyme was quenched by HCl. The product amount was quantified by HPLC after derivatization with OPA. **(B)** HEPES buffer (pH 7.0, 100 mM), *rac*-**3a** (5 mM) and purified AAc (80 μg mL^−1^) with/without NAAAR (300 μg mL^−1^) with different combinations of metal additive (0.2 mM CoCl_2_, 0.2 mM MnCl_2_, and 5 mM FeSO_4_). After 2 h reaction at 25°C, the enzyme was quenched by 2 M HCl. The formation rate was determined by HPLC after derivatization with OPA.

An important issue in the establishment of enzymatic cascade reactions is the mutual compatibility toward the reaction conditions of each enzyme. This regards mostly pH and temperature, but also the inactivation by cofactors or the metal ions required by the different enzymes. AAc, NAAAR and BtDO are all metal-dependent enzymes. The activities of NAAR and AAc are enhanced by the addition of Co^2+^, and BtDO requires Fe^2+^ as a cofactor. Therefore, activity tests in the conversion of methionine were performed in the presence of different metal ions. The tolerance of the iron-dependent BtDO toward cobalt was investigated first. L-methionine (*S*)-**1a** was used as a model substrate and the reaction was performed with different concentrations of cobalt. Concentration of Co^2+^ higher than 0.4 mM clearly inhibited BtDO (Figure 1A). Thus, the best concentration of cobalt ion regarding the activity of BtDO was determined as 0.2 mM.

The compatibility of NAAAR and AAc toward the reaction conditions of the oxyfunctionalization by BtDO is an important prerequisite for the cascade reaction. *In situ* NMR experiments showed that NAAAR is active in a mixture of Co^2+^ and Fe^2+^ (Table 2). Figure 1B shows the effect of different metal ions on AAc and the combination of NAAAR and AAc. Without NAAAR, AAc hydrolyzes the (*S*)-enantiomer and leaves the (*R*)-enantiomer unreacted, leading to 50% maximal conversion. Upon addition of NAAAR, the conversion of the D-amino acid to the L-configuration increases the total conversion. As already seen in the *in situ* NMR experiments, NAAAR tolerates Fe^2+^ but is slightly inhibited. From the inhibition studies of BtDO and NAAAR, a concentration of 0.2 mM Co^2+^ and 0.5 mM Fe^2+^ was assumed to be the best compromise between activation and inhibition of NAAAR and BtDO. Despite a certain extent of inhibition, both enzymes show an acceptable activity under these conditions.

**Table 2:** **Effect of metal additive on racemization activity of NAAAR**.

**Metal additive**	**Conversion (%)**
Without metal	27
0.2 mM Co^2+^	92
0.2 mM Mn^2+^	85
0.5 mM Fe^2+^	45
0.5 mM Fe^2+^, 0.2 mM Co^2+^	94
0.5 mM Fe^2+^, 0.2 mM Mn^2+^	79

*The reaction comoponents were shown below: Potassium phosphate buffer (pH 7.0, 100 mM), rac-**3a** (5 mM) and purified NAAAR (300 μg mL^−1^) with different metal additive dissolved in deuterium oxide. After 2 h reaction at 25°C, the enzyme was quenched by heat. The conversion rate was determined by measuring the H-D replacement of the α-proton in ^1^H-NMR*.

### Sequential and simultaneous combination of DKR and hydroxylation

**Figure 2:**
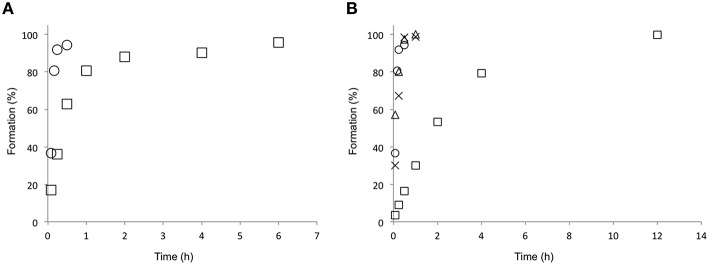
**Combination of AAc and NAAAR for the DKR of ***N***-acetyl-DL-methionine to L-methionine (Scheme 2A)**. **(A)** The effect of reaction temperature on the DKR of *N*-acetyl-DL-methionine (*rac*-**3a**). HEPES buffer (pH 7.0, 100 mM), *rac*-**3a** (10 mM), CoCl_2_ (0.4 mM) AAc (80 μg mL^−1^), and NAAAR (300 μg mL^−1^). The formation rates at 25 and 40°C are represented as square and circle, respectively. **(B)** The substrate scope of NAAAR and AAc cascade reaction. The reaction components were same as mentioned **above** with different substrates (*rac*-**3a-d**). The reaction temperature was 40°C. The formation rate of **1a**, **1b**, **1c**, and **1d** are represented as circle, square, triangle, and cross, respectively.

AAc and NAAAR were combined in the same reaction pot and the DKR was performed with *rac*-**3a** as a substrate. The optimal temperature of BtDO was reported as 25°C (Hibi et al., 2011). Nevertheless, AAc and NAAAR have been reported to work excellently at medium to high temperatures (40–70°C; Cho et al., 1987; Tokuyama, 2001). This makes a sequential cascade approach possible. After the synthesis of the L-amino acid at 40°C (Scheme 2A) the reaction mixture was cooled down to 25°C and BtDO and its required cofactors were added for the stereoselective oxidation (Scheme 2B). In contrast, a simultaneous cascade approach would be required to run at the optimal reaction temperature for BtDO. The combination of AAc and NAAAR was therefore investigated at 25 and 40°C, and over 90% conversion was achieved within 4 and 0.5 h, respectively (Figure 2A). Furthermore, the substrate scope of DKR (AAc and NAAAR) toward *N*-acetyl-DL-methionine (**3a**), -leucine (**3b**), -norvaline (**3c**), and -norleucine (**3d**) was also investigated (Figure 2B and Table 3). While the reaction rate of **3b** was slow due to the low activity of AAc and NAAAR, the combined reaction achieved full conversion toward other substrates within 1 h. This led to the next sequential cascade reaction step producing diastereomerically pure hydroxy amino acids and methionine sulfoxide.

**Table 3:** **The substrate scope of dynamic kinetic resolution via NAAAR and AAc**.

**Substrates**	**Products**	**Time (h)**	**Formation (%)^a^**
3a	1a	1	93
3b	1b	12	99
3c	1c	0.5	97
3d	1d	0.5	98

a *The product formation was determined by HPLC analysis*.

### Comparison of sequential and simultaneous cascade reactions

**Figure 3:**
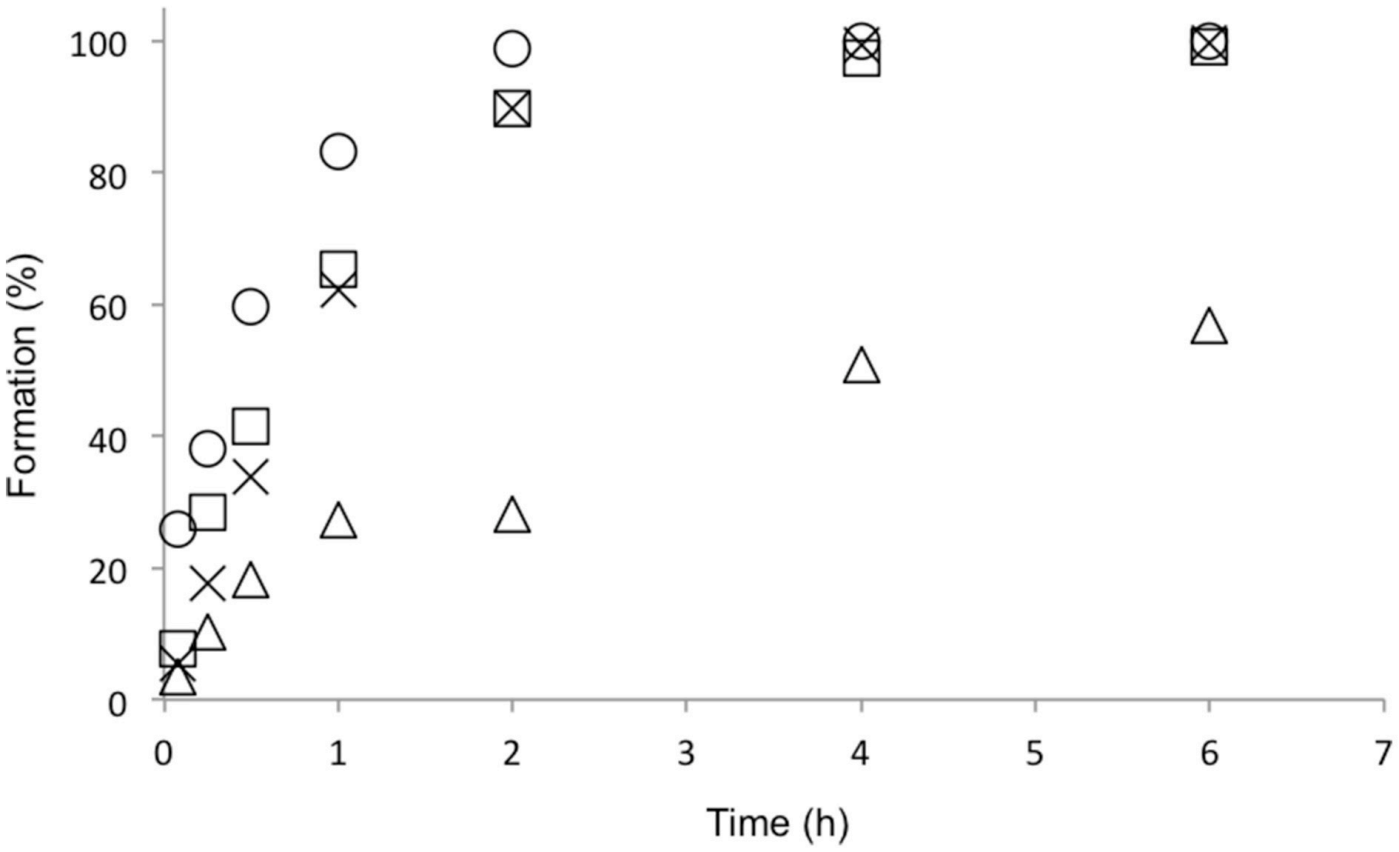
**Formation of oxyfunctionalized amino acids after a sequential cascade reactions using BtDO (Scheme 2B)**. The diagram shows the time course of the second step, the oxyfunctionalization by BtDO.: HEPES buffer (pH 7.0, 100 mM), the reaction products from DKR, α-ketoglutarate (10 mM), ascorbic acid (10 mM), CoCl_2_ (0.2 mM), FeSO_4_ (0.5 mM), and purified BtDO (1.0 mg mL^−1^) The reaction temperature was 25°C. The formation rates of 2a, 2b, 2c, and 2d are represented as circle, square, triangle, and cross, respectively.

The sequential reaction cascade with a first racemization and simultaneous regioselective hydrolysis of *N*-acetylamino acids at 40°C (Scheme 2A) and then a subsequent oxyfunctionalization at 25°C (Scheme 2B) could be shown with several racemic *N*-acetylamino acids. Figure 3 and Table 4 shows the time course of the oxyfunctionalization by BtDO. The substrate spectrum of the dioxygenase is an important factor. While several branched chain amino acids with a moderately long side chain were converted smoothly, norvaline was converted much slower than the others. Nevertheless, several amino acid dioxygenases are available for the identification of fast-reacting enzymes for an impressive number of amino acids (Smirnov et al., 2012). Using *N*-acetyl-DL-norleucine **3d**, we detected two different hydroxy amino acids in the product mixture (Figure S1). This is consistent with the report by Hibi et al. (2011) explaining that BtDO catalyzes γ- and δ-hydroxylation toward L-norleucine **1d**.

**Table 4:** **Oxyfunctionalizations of ***N***-acetyl-DL-amino acids after sequential cascade reactions**.

**Substrates**	**Products**	**Time (h)**	**Formation (%)^a^**
3a	2a	2	98
3b	2b	4	97
3c	2c	6	47
3d	2d	4	99

a *Formation of oxyfunctionalized amino acids were monitored by HPLC analysis*.

**Figure 4:**
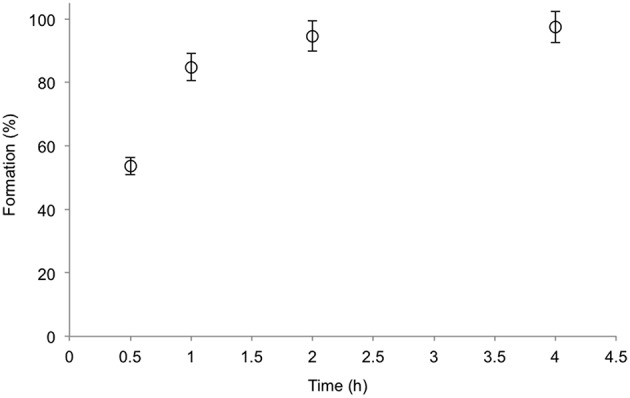
**Formation of L-methionine-(***S***)-sulfoxide (95%***de***) by a three enzyme one-pot cascade reaction using NAAAR, AAc, and BtDO (Scheme 2)**. HEPES buffer (pH 7.0, 100 mM), *rac*-3a (5 mM), α-ketoglutarate (10 mM), ascorbic acid (10 mM), CoCl_2_ (0.2 mM), FeSO_4_ (0.5 mM), purified AAc (80 μg mL^−1^), NAAAR (300 μg mL^−1^), and BtDO (1.0 mg mL^−1^). The temperature was 25°C. The amount of product was quantified by HPLC with OPA derivationzation.

Formation of L-amino acids and stereoselective oxyfunctionalization can also efficiently be combined. At 25°C, a mixture of NAAAR, AAC, and BtDO produced L-methionine-(*S*)-sulfoxide (*S*,*S*)-**2a** with 97% yield after 4 h (Figure 4). The (*S*,*S*)-diastereomer was formed in 95% *de*. The slightly less diastereomeric excess can be explained by a spontaneous oxidation of (*S*)-**1a**.

## Conclusions

Cascade reactions represent an exciting development in enzyme catalysis. While conducting natural pathways *in vitro* is straightforward, the assembly of catalysts from different organisms to artificial enzyme cascades is often challenging. We have shown that the combination of stereoselective oxidation of racemic *N*-acetylamino acids coupled with enzymatic racemization can be used to increase the yield to a theoretical 100%. This study shows the mutual inhibition by metal cofactors can be reduced to an acceptable extent by reaction optimization. By using an enantioselective *N*-acetylamino acid acylase for the generation of the stereocenter bearing the amino group, we were able to conduct the cascade in a step-wise fashion. Compared with “direct” dynamic kinetic reactions of racemic amino acids into oxidized products using AAR and BtDO, this three-enzyme cascade reaction allows to separate the oxyfunctionalization step from the DKR part. This made it possible to set the ideal reaction temperature for each enzyme. In the stereoselective oxidation of L-methionine, we were then able to demonstrate the feasibility of a simultaneous cascade leading from inexpensive racemic *N*-acetylamino acids directly to diastereomerically pure hydroxylated and sulfoxy products. As several hydroxylases with δ- and γ-regioselectivity are available, the approach can be applied for the synthesis of a large series of oxyfunctionalized amino acids (Smirnov et al., 2012). BtDO shows a rather low reaction rate, which is typical for enzymatic oxyfunctionalization reactions. After demonstrating the feasibility of the cascade concept, future research will focus on conducting the cascade in a whole-cell system.

## Author contributions

AM and JM carried out the cloning and functional expression of a bacterial dioxygenase and its purification and characterization. JE contributed to the conception and design of the work and carried out the cloning and expression of acylase and racemase, the establishment of multi-enzyme cascade reactions, the product characterization and chiral analytics. RK devised the work. JE and RK wrote the manuscript, which was critically revised by all authors. All authors read and approved the final manuscript.

## Funding

This work was funded by the Mercator Research Center Ruhr (Pr-2013-0010).

### Conflict of interest statement

The authors declare that the research was conducted in the absence of any commercial or financial relationships that could be construed as a potential conflict of interest.

## Abbreviations

BtDO: Isoleucine dioxygenase from *B. thuringiensis*
AAc: Aminoacylase from *G. thermoglucosidasius* DSM2542
NAAAR: *N*-Acylamino acid racemase mutant G291D/F323Y from *Amycolatopsis* sp. Ts1-60
OPA: *o*-Phtalaldehyde
HPLC: High-performance liquid chromatography.
