# Supplementary material for: A Multi-Enzymatic Cascade Reaction for the Stereoselective Production of γ-Oxyfunctionalyzed Amino Acids
Source: Front Microbiol. 2016 Apr 7;7:425. doi: 10.3389/fmicb.2016.00425 (PMC4823265; doi:10.3389/fmicb.2016.00425)
Supplement: Supplementary file 1 [file DataSheet1.DOCX]

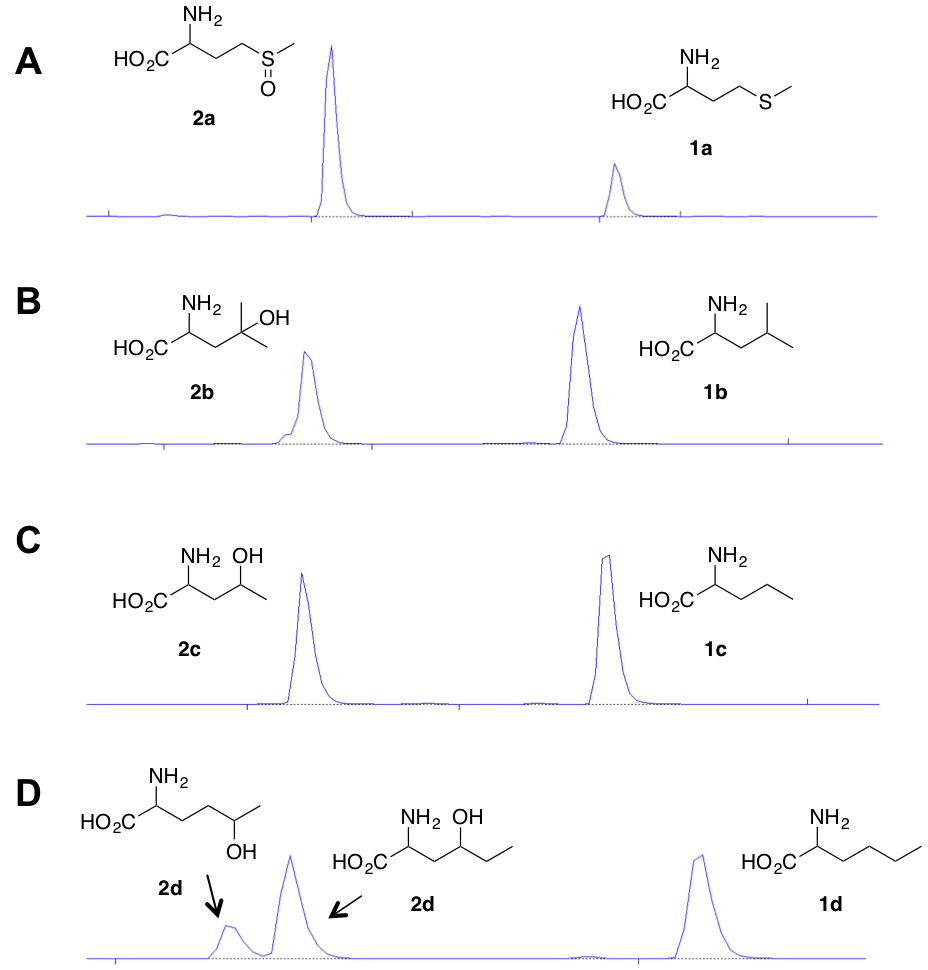


**Figure S1 | HPLC chromatograms of sequential cascade reaction products.** 1 h reaction products of *rac*-**3a** (A), -**3c** (C) and -**3d** (D) and 6 h reaction product of *rac*-**3b** (B)

*aac* gene sequence

5’- ATGGGCAGCAGCCATCATCATCATCATCACAGCAGCGGCCTGGTGCCGCGCGGCAGCCATATGGCTAGCATGACCAATGAAGAGATCAAACGGCTTGTCGATGAAGTGAAAGAGGAAGTGATTGCTTGGCGCCGTCATTTGCATGCAAATCCTGAATTGTCTTTTCAGGAAGAAAAAACGGCGCAGTTTGTTTATGAGACATTGCAGTCGTTTGGAAATCTGGAGATTTCACGGCCAACCAAAACAAGCGTGATGGCGCGGCTGATCGGGCCTCAGCCGGGGAGAGTGGTGGCGATTCGCGCCGATATGGACGCGTTGCCGATTCAAGAAGAAAACACGTTTGCATTTGCTTCGAAAAATCCCGGCGTGATGCATGCGTGCGGGCATGACGGACATACGGCGATGCTTTTAGGAACGGCGAAAATTCTTTCTCAGCTGCGCGATCAAATTAAAGGGGAAATCCGTTTTCTTTTCCAGCATGCCGAAGAGTTGCATCCAGGCGGTGCAGAAGAAATGGTGCAGGCCGGTGTCATGGACGGCGTTGATGTCGTGATCGGCACTCATCTTTGGTCGCCGCTTGAGCGCGGCAAAATTGGCATTGTGTACGGACCGATGATGGCGGCCCCAGACCGCTTTTTCATCCGCATCCATGGCAAAGGCGGACACGCGGCGCTGCCGCATCAAACGATTGATGCGATTGCGGTCGGCGCCCAAGTGGTAACCAACTTGCAGTATATTGTTTCGCGCAATGTTGATCCGCTTGAACCGCTGGTCGTGTCGGTAACGCAATTTGTCGCAGGAACGACGCATAACGTCATTCCGGGAAGCGTTGAAATTCAAGGAACGGTGCGCAGTTTCGATGAGACACTGAGAAAAAGTGTGCCGAAATTAATGGAACGGATTATTAAAGGCATAACCGAAGCGCATGGCGCGACATATGAATTTGAATTTGAATACGGCTACCGTCCGGTCATTAACAATAACGAGGTTACCCGCGTGATTGAGGAAACGGTGCGCGAAGTATTTGGGGAGGAAGCGGTTGACCATATAAAGCCAAATATGGGCGGCGAAGATTTTTCCGCATTCCAGCAAAAAGCCCCGGGCAGCTTCTTCTATGTCGGTGCAGGAAACAAAGAAAAAGGCATCGTCTATCCGCATCATCATCCACGCTTTACCATCGACGAAGATGCGTTAGAAATTGGCGTGCGCCTGTTTGTCCATGCGACGTTTAAATTATTGGCGGAAGCGTCATAA

-3’

*ido* gene sequence

5’-

ATGAAAATGAGTGGCTTTAGCATAGAAGAAAAGGTACATGAATTTGAATCTAAAGGGTTTCTTGAAATCTCAAATGAAATCTTTTTACAAGAGGAAGAGAATCATAGTTTATTAACACAAGCACAGTTAGATTATTATAATTTGGAAGATGATGCGTACGGTGAATGCCGTGCTAGATCTTATTCAAGGTATATAAAGTATGTTGATTCACCAGATTATATTTTAGATAATAGTAATGATTACTTCCAATCTAAAGAATATAACTATGATGATGGCGGGAAAGTTAGACAGTTCAATAGCATAAATGATAGCTTTTTGTGTAATCCTTTAATTCAAAATATCGTGCATTTCGATACTGAGTTTGCATTTAAAACAAATATAATAGATAAAAGTAAAGATTTAATTATAGGCTTACATCAAGTAAGATATAAAGCTACTAAAGAAAGACCATCTTTTAGTTCACCTATTTGGTTACATAAAGATGATGAACCAGTAGTGTTTTTACACCTTATGAATTTAAGTAATACAGCTATCGGCGGAGATAATTTAATAGCTAATTCTCCTCGGGAAATTAATCAGTTTATAAGTTTGAAGGAGCCGTTAGAAACTTTAGTATTTGGACAAAAGGTCTTCCATGCCGTAACGCCACTTGGAACAGAATGTAGTACGGAGGCTTTTCGTGATATTTTATTAGTAACATTTTCTTATAAGGAGACAAAACTCGAGCACCACCACCACCACCACTGA

-3’

*naaar* gene sequence

5’-

ATGAAACTGAGCGGTGTTGAACTGCGTCGTGTTCAGATGCCGCTGGTTGCACCGTTTCGTACCAGCTTTGGCACCCAGAGCGTTCGTGAATTACTGCTGCTGCGTGCAGTTACACCGGCAGGCGAAGGTTGGGGTGAATGTGTTACCATGGCAGGTCCGCTGTATAGCAGCGAATATAATGATGGTGCAGAACATGTGCTGCGTCATTATCTGATTCCGGCACTGCTGGCAGCAGAAGATATTACCGCAGCAAAAGTGACACCGCTGCTGGCAAAATTCAAAGGTCATCGTATGGCAAAAGGTGCACTGGAAATGGCAGTTCTGGATGCCGAACTGCGTGCACATGAACGTAGCTTTGCAGCAGAACTGGGTAGCGTGCGTGATAGCGTTCCGTGTGGTGTTAGCGTTGGTATTATGGATACCATTCCGCAGCTGCTGGATGTTGTTGGTGGTTATCTGGATGAAGGTTATGTGCGCATTAAACTGAAAATTGAACCGGGTTGGGATGTTGAACCGGTGCGTGCGGTTCGTGAACGTTTTGGTGATGATGTGCTGCTGCAGGTTGATGCAAATACCGCATATACCCTGGGTGATGCACCTCAGCTGGCACGTCTGGATCCGTTTGGTCTGCTGCTGATTGAACAGCCGCTGGAAGAAGAGGACGTTCTGGGTCATGCGGAACTGGCACGCCGTATTCAGACCCCGATTTGCCTGGATGAAAGCATTGTTAGCGCACGTGCAGCAGCCGATGCAATTAAACTGGGTGCAGTTCAGATTGTGAACATTAAACCGGGTCGTGTGGGAGGCTATCTGGAAGCACGTCGTGTGCATGATGTTTGTGCAGCCCATGGTATTCCGGTTTGGTGTGGTGATATGATTGAAACCGGTCTGGGTCGTGCAGCAAATGTTGCACTGGCAAGCCTGCCGAATTTTACCCTGCCTGGTGATACCAGCGCAAGCGATCGTTATTACAAAACCGATATTACAGAACCGTTTGTGCTGAGTGGTGGTCATCTGCCGGTTCCGACCGGTCCGGGTCTGGGTGTTGCTCCGATCCCGGAACTGCTGGACGAAGTTACCACCGCAAAAGTTTGGATTGGTAGCTAACTCGAGCACCACCACCACCACCACTGA

-3’References

Baxter, S., Royer, S., Grogan, G., Brown, F., Holt-Tiffin, K. E., Taylor, I. N., Fotheringham, I. G., Campopiano, D. J. (2012). An improved racemase/acylase biotransformation for the preparation of enantiomerically pure amino acids. *J. Am. Chem. Soc, 134*(47), 19310-19313.

Cho, H.-Y., Tanizawa, K., Tanaka, H., and Soda, K. (1987). Thermostable aminoacylase from Bacillus thermoglucosidius: Purification and characterization. *Agric. Biol. Chem., 51*(10), 2793-2800.

Hibi, M., Kawashima, T., Kodera, T., Smirnov, S. V., Sokolov, P. M., Sugiyama, M., Shimizu, S., Yokozeki, K., and Ogawa, J. (2011). Characterization of *Bacillus thuringiensis* L-isoleucine dioxygenase for production of useful amino acids. *Appl. Environ. Microbiol., 77*(19), 6926-6930.
